# Pediatric Injury Surveillance From Uncoded Emergency Department Admission Records in Italy: Machine Learning–Based Text-Mining Approach

**DOI:** 10.2196/44467

**Published:** 2023-07-12

**Authors:** Danila Azzolina, Silvia Bressan, Giulia Lorenzoni, Giulia Andrea Baldan, Patrizia Bartolotta, Federico Scognamiglio, Andrea Francavilla, Corrado Lanera, Liviana Da Dalt, Dario Gregori

**Affiliations:** 1 Department of Environmental and Preventive Sciences University of Ferrara Ferrara Italy; 2 Department of Women's and Children's Health University of Padova Padua Italy; 3 Unit of Biostatistics, Epidemiology and Public Health Department of Cardiac, Thoracic, Vascular Sciences, and Public Health University of Padova Padua Italy

**Keywords:** machine learning, pediatrics, child and adolescent health, text mining, injury, death, surveillance, pediatric admission, hospitalization, patient record, unintentional injury, emergency department, emergency, epidemiological surveillance

## Abstract

**Background:**

Unintentional injury is the leading cause of death in young children. Emergency department (ED) diagnoses are a useful source of information for injury epidemiological surveillance purposes. However, ED data collection systems often use free-text fields to report patient diagnoses. Machine learning techniques (MLTs) are powerful tools for automatic text classification. The MLT system is useful to improve injury surveillance by speeding up the manual free-text coding tasks of ED diagnoses.

**Objective:**

This research aims to develop a tool for automatic free-text classification of ED diagnoses to automatically identify injury cases. The automatic classification system also serves for epidemiological purposes to identify the burden of pediatric injuries in Padua, a large province in the Veneto region in the Northeast Italy.

**Methods:**

The study includes 283,468 pediatric admissions between 2007 and 2018 to the Padova University Hospital ED, a large referral center in Northern Italy. Each record reports a diagnosis by free text. The records are standard tools for reporting patient diagnoses. An expert pediatrician manually classified a randomly extracted sample of approximately 40,000 diagnoses. This study sample served as the gold standard to train an MLT classifier. After preprocessing, a document-term matrix was created. The machine learning classifiers, including decision tree, random forest, gradient boosting method (GBM), and support vector machine (SVM), were tuned by 4-fold cross-validation. The injury diagnoses were classified into 3 hierarchical classification tasks, as follows: injury versus noninjury (task A), intentional versus unintentional injury (task B), and type of unintentional injury (task C), according to the World Health Organization classification of injuries.

**Results:**

The SVM classifier achieved the highest performance accuracy (94.14%) in classifying injury versus noninjury cases (task A). The GBM method produced the best results (92% accuracy) for the unintentional and intentional injury classification task (task B). The highest accuracy for the unintentional injury subclassification (task C) was achieved by the SVM classifier. The SVM, random forest, and GBM algorithms performed similarly against the gold standard across different tasks.

**Conclusions:**

This study shows that MLTs are promising techniques for improving epidemiological surveillance, allowing for the automatic classification of pediatric ED free-text diagnoses. The MLTs revealed a suitable classification performance, especially for general injuries and intentional injury classification. This automatic classification could facilitate the epidemiological surveillance of pediatric injuries by also reducing the health professionals’ efforts in manually classifying diagnoses for research purposes.

## Introduction

Unintentional injury is the leading cause of morbidity and mortality in children worldwide [1]. An injury is defined as tissue damage that occurs secondary to acute exposure to physical agents (eg, thermal, kinetic, chemical, electrical, or electrical energy or water) or chemicals (eg, poisoning). An injury can be fatal or nonfatal and can occur unintentionally or as a result of purposeful acts of harm (intentional) [2]. Unintentional injuries can be prevented or controlled because they are potentially understandable and predictable [3].

In 2013, 15.4% of 2.6 million unintentional injuries worldwide involved a fatal outcome for children between 1 and 14 years of age [1]. In Europe, 42,000 children and adolescents aged 0-19 years died of unintentional injuries in 2004 [4]. Moreover, a considerable number of children may incur some form of disability as a result of injury, often with lifelong consequences [5]. Decreasing the injury burden is the main challenge for child and adolescent public health policies over the next century [6]. For this reason, public health departments must pay more attention to the problem to implement prevention policies [6].

Injury surveillance is made difficult by a series of logistic and structural challenges, the most important of which is the accurate coding of injury mechanisms, products involved, types of injury, and body parts involved, given that emergency department (ED) admission and discharge records are largely based on narrative free-text notes [7]. Injury surveillance integrated with timely data dissemination is crucial for planning and evaluating prevention policies [8] and quantifying injury burden and related risk factors [9,10].

In Italy and other European and newly developed countries [11], narratives and free-text records are standard tools for reporting patient diagnoses. Automatic classification of such free-text information using machine learning techniques (MLTs) would be a powerful tool to improve injury surveillance [12].

This is true, especially for the ED, where physicians and medical personnel often face stressful situations from a clinical and management perspective [13]. Within this general framework, it could be promising to provide an automated MLT-based system aimed at facilitating free-text diagnosis encoding, by also limiting an additional burden for the overwhelmed medical staff. This MLT-based system could be tailored for research and epidemiological surveillance purposes. Furthermore, this surveillance system could be promising for pediatric injury surveillance purposes because most of the incidents that occur on the ground are referred to such departments, especially large pediatric EDs [13].

The literature over the past 10 years indicates an increasing interest in the automated categorization of free-text diagnoses due to the increased availability of documents in digital form [14,15]. Automatic MLT classifiers can learn (during the training phase) from a set of manually classified documents with complex free-text lexical patterns. A properly trained MLT tool can categorize a free-text record into its corresponding class. The advantages of this approach over manual methods are efficiency and saving time (in terms of expert labor) for free-text classification [15].

Statistical text mining methods can also be useful tools to classify electronic ED admission records and properly identify unintentional injury events [15].

This study represents, to our knowledge, the first effort in the literature in proposing an automatic injury classification system based on the free-text data of pediatric ED diagnoses. We propose this algorithm to facilitate injury epidemiological surveillance. The system is aimed at limiting the burden of health care professionals, who are overburdened by patient care and management tasks, in manually classifying diagnoses for epidemiological research.

## Methods

### Data Selection

**Figure 1:**
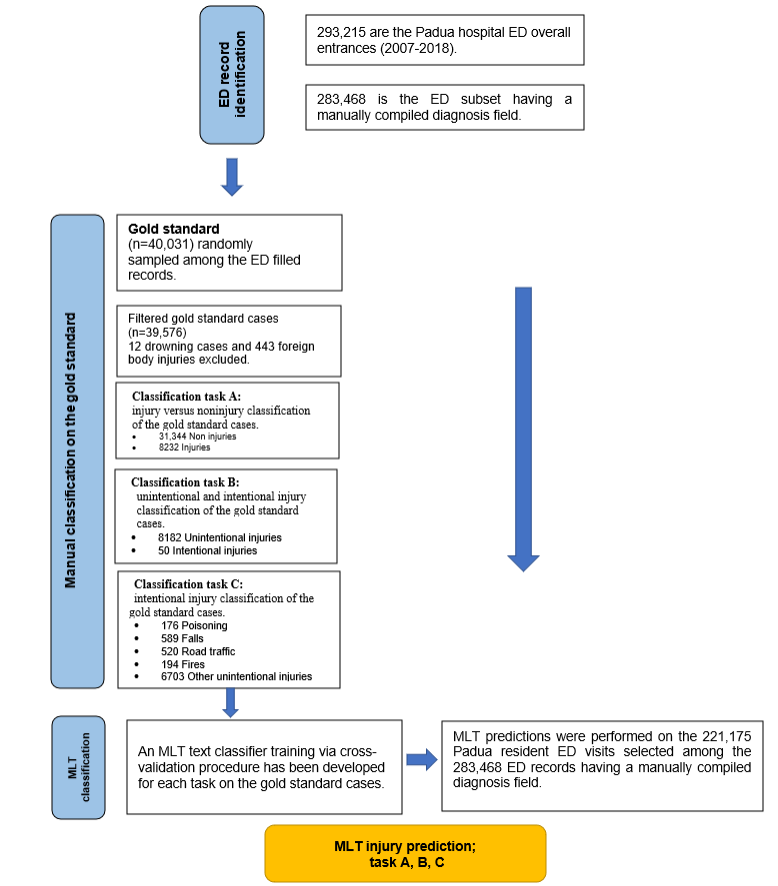
Study flowchart—emergency department (ED) selection and gold standard identification together with manual injury classification procedure. Machine learning technique (MLT) cross-validation and prediction procedures for tasks A, B, and C are represented by the dark grey box.

The study included 283,468 pediatric ED records with a filled discharge diagnosis field among 293,215 records [16] from the local electronic medical record system of Padova University Hospital in Northeast Italy between 2007 and 2018 (Figure 1).

The average ED annual workload is approximately 25,000 visits. The upper age limit to access the pediatric ED is 15 years. A higher and more variable age limit applies to children followed by the Department of Pediatrics for chronic illnesses. The Padova Hospital Pediatrics ED is characterized by high patient turnover with an average hospitalization time of 4-5 days. The number of admissions after ED access is approximately 850 per year.

### Ethics Approval

The study was conducted according to the guidelines of the Declaration of Helsinki and approved by the Ethics Committee of Azienda Ospedaliera of Padova (Hospital Ethics Committee; 0022925) on April 8, 2021. The analysis is carried out on observational data of a secondary nature; however, patients signed a consent form to allow the data to be used for scientific purposes at the time of collection. The records of each patient are kept anonymous with an appropriate identification key excluding personal information. No Compensation has been provided for subjects involved in the research.

### Learning Algorithm for Epidemiological Surveillance

**Figure 2:**
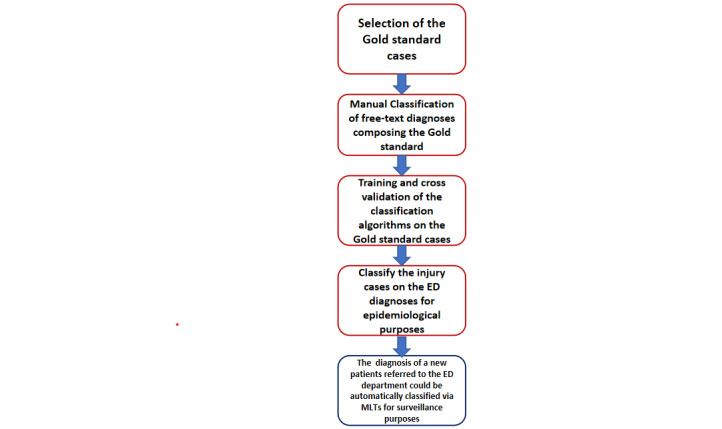
Flowchart of learning algorithm development for injury epidemiological surveillance. ED: emergency department; MLT: machine learning technique.

The definition of the free-text classification algorithm and its use for epidemiological surveillance purposes consisted of several phases, as reported in Figure 2. These phases are the following:

1. A training set was defined as a gold standard and was composed of a random sample of ED diagnoses.
2. The gold standard diagnoses were manually classified by an expert physician into injury versus noninjury (task A), intentional versus unintentional injury (task B), and type of unintentional injury (task C), according to the World Health Organization (WHO) classification of injuries.
3. The preprocessed and manually classified gold standard cases were used to train the MLTs algorithms in classifying the diagnoses according to tasks A, B, and C. Several algorithms were considered to define the MLT tools; the most performing algorithms would be considered for the predictive tool definition.
4. The trained tool served to automatically classify the remaining ED diagnoses by providing a proof of concept of the injury epidemiology in the geographical area referring to the Padua ED center.
5. Once optimal algorithms were defined, they could be used to classify diagnoses on a new ED referral pediatric patient by defining an automated epidemiological surveillance system.

### Gold Standard Definition

A randomly extracted subset of 40,031 ED records was manually classified (Figure 1) by a clinician for the following 3 classification tasks:

1. Injury or noninjury events, characterized as classification task A.
2. Intentional or unintentional injury events as classification task B (performed on diagnoses classified as injury in classification task A).
3. Unintentional injury category as classification task C (performed on records classified as unintentional injury in classification task B).

In this study, unintentional injury ED records were classified based on the WHO [17] classification of 5 types of unintentional injuries: road traffic injuries, poisoning, falls, fires, and drowning. We added a sixth category of unintentional injury exclusive of the WHO categories called “other unintentional injury.” In addition, the WHO classes represented by fewer than 15 records were not considered for cross-validation of the automatic MLT classifier.

Foreign body or choking injury events were also excluded because they constitute a separate epidemiological category conducive to purpose-specific studies [18].

### Data Preprocessing

Free-text diagnoses were preprocessed by removing punctuation, as reported in the literature [19], stop words, white spaces, and numbers, leaving only word stems. All words were converted to the lowercase font.

After cleaning the text corpus, the free-text data were represented via a document-term matrix (DTM) that represented the diagnostic text data in the form of a matrix. The rows of the matrix reported the sentence for the single diagnosis, and the columns of the matrix represented the single word that composed the sentence. The DTM was filled by weighting each word term with the inverse of its frequency.

The most frequent words in the ED free-text records were also reported considering the unprocessed DTM in manually classified gold standard cases.

Different MLT algorithms were trained and tuned to classify injury diagnoses, as follows:

- The decision tree (DT), random forest (RF), and gradient boosting method (GBM) tree-based models.
- The support vector machine (SVM) method served as a comparison tool against the tree-based models.

### MLT Classifier Cross-Validation

The filtered manual classification of 39,576 cases (Figure 1) served as the gold standard for training and cross-validating (4-fold) the MLT tool. In the literature, it is documented that 4- or 5-fold cross-validation is appropriate to minimize the SD accuracy estimate of a tuned model [20].

### Tree-Based Methods and the Comparator

#### Decision Trees (DTs)

DTs are classification or regression models based on a top-down methodology in which, starting from the root node, binary splits of data are generated until a certain criterion is satisfied. The classification error rate has been considered as the fraction of training observations in a particular tree partition that doesn’t belong to the most widely occurring class [21].

The DT is a classification method that in several cases suffers from overfitting; for this reason, ensemble methods are provided in the literature. For example, bagging or bootstrap aggregation is a technique for reducing the variance of an estimated prediction function. Bagging seems to work especially well with high variance and low-bias procedures, such as trees [22].

#### Random Forest (RF)

Rf is a modification of the bagging method that constructs a large collection of poorly correlated trees and then calculates the average. In many problems, the performance of RF is high; RFs are also easy to train and regularize. As a result, they have become quite popular [23]. The RF tree-based algorithm involves the computation of hundreds to thousands of DTs and merges them to increase the generalizability of the model. The DT combination essentially takes the form of an ensemble method. Weak learner (or single DT) pooling is used as a strategy to obtain more powerful learners [22].

#### Gradient Boosting Method (GBM)

The GBM is based on sequential boosting improvements of weak classifiers (high bias and low variance). The GBM sequentially adds one classifier at a time so that the next classifier is trained to improve the previously trained DT. In contrast, the RF algorithm trains each classifier independently of the others [22].

#### Support Vector Machine (SVM) as the Comparator

The main objective of the SVM algorithm is to find an optimal hyperplane of a feature space of N dimensions (where N is the number of features) that distinctly classifies the data points into a binary partition [24]. Several hyperplanes may separate the resulting classes of data points. The SVM algorithm considers the hyperplanes that maximize the margin (the distance between the data points of the classes). The SVM algorithm was selected as a comparator for tree-based algorithms. The kernel hyperplane approach has been considered for the computation.

### Classification Tasks

Three classification tasks were considered for the analysis, as follows:

- Classification of injury versus noninjury events (task A)
- Classification of intentional versus unintentional injury events (task B)
- Classification of unintentional injuries (task C) based on the WHO categorization (poisoning, road traffic, falls, fires and burns, drowning, and other unintentional injuries) [5].

### Performance Evaluation

The performance of the MLT classifiers was evaluated using cross-validated accuracy and Kappa agreement values compared to the gold standard. In particular, the training set represented by the gold standard is one of the largest such sets produced to classify injuries.

For classification scenarios that involved severe class imbalance (where the minority class is represented by less than 15% of the cases), balanced accuracy was reported.

The mean and maximum accuracies computed for all classes that included unintentional injuries in the gold standard records were also calculated. Other performance measures were reported concerning positive and negative predictive values, sensitivity, and specificity.

### MLT Predictions

MLT predictions for classification tasks A, B, and C were calculated for admissions to the ED of children residing in Padua (221,175 records; Figure 1). Subsequently, Poisson 95% CIs for injury incidence rates over the Padova province resident child (aged 0-18 years) population were computed to compare the predictions of the different MLT methods. The person-time was identified in the period of 2007-2018 by considering the official Italian statistic data source ISTAT [25]. The number of cases in the period was estimated using the RF, GBM, DT, and SVM algorithms.

### Synthesis of Data

Summary statistics of the gold-standard case data were reported as follows: continuous data were summarized as first quartile, median, and third quartile; categorical data were reported as percentages and absolute frequencies. Wilcoxon-type tests were performed for continuous variables, and Pearson chi-square test or Fisher exact test, as appropriate, were performed for categorical variables.

The computations were performed using R 3.4.2 (R Foundation for Statistical Computing) [26] with the caret package [27] as a machine learning R interface and the rms package [28] for descriptive and standard statistical analyses.

## Results

### Gold Standard Description

The gold standard set used to train the MLT classifiers (39,576 cases) was composed of 19,659 female and 19,917 male individual admissions (Table 1). The sample was mainly composed of Italians, and 33,474 (85%) gold standard cases were aged between 1 month and 15 years (Table 1).

Injury events were mainly represented by Italian male children between 6 and 15 years of age (Table 1). Among the 8232 injuries in Table 1, only 50 (0.6%) cases were intentional injuries.

Manually classified WHO unintentional injury drowning cases were not considered in the analyses because there were only 12 such cases (Figure 1). Falls and road traffic injuries were the main types of unintentional cases in the gold standard set (Table 1).

**Table 1:** Characteristics of the gold standard cases. Continuous data are reported as medians (first and third quartiles); categorical data are reported as percentages and absolute frequencies. Wilcoxon-type tests were performed for continuous variables; Pearson chi-square test or Fisher exact test, as appropriate, were performed for categorical variables.

Characteristics		Noninjury (N=31,344), n (%)	Injury (N=8232), n (%)	Overall (N=39,576), n (%)	*P* value
**Gender**					<.001
	Female	15,762 (50)	3897 (47)	19,659 (50)	
	Male	15,582 (50)	4335 (53)	19,917 (50)	
**Age**					<.001
	1-28 days	4056 (13)	103 (1)	4159 (11)	
	29 days-1 year	6035 (19)	702 (9)	6737 (17)	
	1-3 years	5293 (17)	1388 (17)	6681 (17)	
	4-5 years	5270 (17)	1387 (17)	6657 (17)	
	6-10 years	4720 (15)	1945 (24)	6665 (17)	
	11-15 years	4170 (13)	2564 (31)	6734 (17)	
	≥16 years	1800 (6)	143 (2)	1943 (5)	
**Nationality**					<.001
	Other countries	14,875 (47)	3395 (41)	18,270 (46)	
	Italian	516,469 (53)	4837 (59)	21,306 (54)	
**Season**					<.001
	Spring	7590 (24)	2114 (26)	9704 (25)	
	Summer	7540 (24)	2381 (29)	9921 (25)	
	Autumn	7783 (25)	2175 (26)	9958 (25)	
	Winter	8431 (27)	1562 (19)	9993 (25)	
**Manual classification of events**					<.001
	Noninjury	31,344 (100)	—^a^	31,344 (79)	
	Injury (intentional)	—	50 (1)	50 (0)	
	Injury (unintentional: poisoning)	—	176 (2)	176 (0)	
	Injury (unintentional: falls)	—	589 (7)	589 (1)	
	Injury (unintentional: road traffic)	—	520 (6)	520 (1)	
	Injury (unintentional: fires and burns)	—	194 (2)	194 (0)	
	Injury (unintentional: other)	—	6703 (81)	6703 (17)	

^a^Not applicable.

### Free-Text Diagnosis Description

The free-text diagnosis field of the gold standard cases was preprocessed, and the DTM was synthesized based on word occurrence. A manual frequency evaluation of noninjury diagnoses found the most frequent words were “high,” “respiratory,” “tract,” “inflammatory,” and “fever,” whereas the most frequent words in injury diagnoses were “skull,” “trauma,” “wound,” “fracture,” and “hand” (Figure S1 in Multimedia Appendix 1).

Regarding the unintentional injury classes (Figure S2 in Multimedia Appendix 1), “trauma” was the most frequent word found in falls, road traffic, and other unintentional injury diagnoses.

Burn events were mainly described with hand-related attributes such as “right” and “left.” No drowning events were reported.

The words “ingestion” and “suspected” were mainly associated with poisoning diagnoses.

### MLT Classifier Performance

#### Classification Task A: Injury Versus Noninjury

The average cross-validated accuracy reported as percentages for the different MLT classifiers was greater than 85% in every case and was very similar for the SVM, GBM, and RF models (Table 2).

A high level of agreement among the methods can be found using the kappa measure, which was greater than 79% in every case with the exception of DT (Table 2). In addition, the kappa performance of the RF and SVM methods was very similar.

Most disagreement cases stemmed from a mismatch with the gold standard, and the methods exhibited a high level of consistency in this regard. For example, the percentage of correctly evaluated injuries was very similar for the SVM and RF disagreement cases (Table S1 in Multimedia Appendix 1).

**Table 2:** Injury versus noninjury classification task comparative cross-validated accuracies and kappa scores of the machine learning technique (MLT) classifiers.

Feature	Random forest	Decision tree	Gradient boosting method	Support vector machine
Overall accuracy (%)	94.09	88.38	94.1	94.14
Overall kappa (%)	81.76	58.66	81.63	81.77
Sensitivity	0.980475	0.993396	0.985065	0.983218
Specificity	0.795633	0.381413	0.7518	0.790492
Positive predictive value	0.945387	0.852819	0.934727	0.944237
Negative predictive value	0.918657	0.941199	0.933117	0.928852

#### Classification Task B: Intentional Versus Unintentional Injury

The balanced accuracy performance was greater than 70% for RF, GBM, DT, and SVM (Table 3).

Considering the other metrics (ie, sensitivity, specificity, negative predictive values, and positive predictive values), the algorithms were able to classify unintentional cases, as the negative predicted values were greater than 99% in every unintentional case. Intentional injuries, in contrast, were misclassified in several cases, and the positive predictive values were less than 2% in every case (Table 3).

**Table 3:** Intentional versus unintentional injury classification task comparative cross-validated performance measures of the machine learning technique (MLT) classifiers.

Feature	Random forest	Decision tree	Gradient boosting method	Support vector machine
Balanced accuracy (%)	71.33	75.63	76.65	70.86
Sensitivity	0.6867	0.7	0.7644	0.64
Specificity	0.7401	0.7787	0.7563	0.7631
Positive predictive value	0.0151	0.018	0.0178	0.0154
Negative predictive value	0.9976	0.9978	0.9982	0.9973

#### Classification Task C: Unintentional Injury Category

The algorithms were trained and tuned on different subclasses of unintentional injuries, and the scores of a balanced accuracy measure were found to be relatively greater and similar for RF, SVM, and GBM, and smaller for DT (Table 4).

For the other metrics, all the algorithms correctly identified poisoning and other unspecified injuries. For these classes, the classification positive predictive values were greater than 60% in every case for the different MLT methods (Table S2 in Multimedia Appendix 1). The metrics reported in Table S2 (Multimedia Appendix 1) also revealed decreased performance for the identification of trauma-related injuries, such as “burns,” “falls,” and “road traffic,” where the sensitivity was less than 15%.

**Table 4:** Unintentional injury classification task (in every case, maximum accuracy is achieved for the poisoning class).

Feature	Random forest	Decision tree	Gradient boosting method	Support vector machine
Maximum balanced accuracy (%)	91.42	81.09	91.63	91.62
Mean balanced accuracy (%)	64.59	59.26	65.07	64.95

### Predicted MLT Injury Incidence Rates

For the 221,175 ED visits studied (Table S3 in Multimedia Appendix 1), the median age was 4 years, and the majority of the children were of Italian nationality (172,577, 78%). The estimated number of injury cases was similar for RF, GBM, and SVM but relatively lower for DT (Table S4 in Multimedia Appendix 1).

The estimated incidence rates of ED entrance for Padova residents (2007-2018) were very similar across the GBM, RF, and SVM methods and slightly lower for DT (Figure S3 in Multimedia Appendix 1).

## Discussion

### Principal Findings

In Italy, unintentional injuries in children have been scarcely investigated. Padova Hospital is an important health center in Northeast Italy, characterized by a high number of daily ED visits due to unintentional injuries. Analysis of the Padova Hospital ED database represents a suitable starting point for the development of a reliable and generalizable epidemiological injury surveillance system.

The free-text classification may improve the epidemiologic surveillance of pediatric ED injuries. However, the manual classification of free-text diagnoses is often time-consuming and requires highly trained clinicians [29]. On the contrary, automated text classification approaches require relatively fixed data sources and can improve the efficiency and timeliness of ED surveillance systems [29].

Several MLT methods are currently used to perform automatic text classification. The methodological comparison of different MLTs is useful to achieve a valid and accurate text classification [30]. This study demonstrates that ensemble tree-based resampling methods (RF and GBM) and SVMs are consistent with each other [31], reporting good classification accuracy [11] over different classification tasks, as corroborated in the literature [12]. DT is known to have a high variance when using training or test sets different from the same data set because it is prone to overfitting. Moreover, the optimal choice of an MLT classifier should be integrated and tailored to gold-standard data characteristics, such as the number of classes, class imbalance, and the correlation structure of predictors. In the literature, ensemble methods (ie, RF and GBM) have been shown to be more robust in relation to these previously mentioned issues [32].

Cross-validation is a useful method that limits overfitting and allows tuning of DT parameters to optimize model accuracy [33]. The best classifier performance in this study was achieved on the task of identifying injury versus noninjury cases in ED visits (task A).

Regarding surveillance, the implications of our results are clear; current injury surveillance systems are largely based on mortality or hospital discharge data [13]. However, thousands of pediatric patients are treated in the EDs and subsequently discharged [13]. In Italy and other European and newly developed countries, ED data often contain narratives and free text to describe patient diagnoses [11]. Thus, an automated ED surveillance system, not requiring additional physician work, would be a suitable tool for comprehensive surveillance of childhood injuries. A negative predictive value of at least 99% was found to identify unintentional injuries; this indicates that there is a high probability that the cases identified as unintentional by this algorithm were unintentional. MLTs are capable of correctly classifying unintentional cases, which are highly prevalent injury events. In the literature [34], unintentional injury ED visits were found nearly 20 times more than intentional injury ED visits in the United States, and the pattern is similar in European countries [35].

The algorithms in this study performed poorly on the identification of intentional events. The reason for this poor performance was due to a lack of intentional injury cases (n=50, 0.6%) in the data [19]. Other methods are needed to develop a more accurate free-text classifier for intentional injury events. Poor performance was also evident in the distinction of trauma-related injuries (eg, falls and road traffic injuries).

MLTs (especially ensemble algorithms) have shown good classification performance in poisoning events. From an epidemiological perspective, poisoning events remain the third most common cause of unintentional injuries in Italy and Europe [4].

Large EDs are important sources of surveillance for pediatric diseases, especially for trauma and injury-related issues, given that most of such events refer to these departments [13]. However, the staff employed in such facilities often work in stressful situations, and the time and human resources to devote to data collection and accurate diagnosis coding may be very limited. In this general framework, our proposed MLT-based tool could facilitate the automatic classification of events for surveillance purposes. Once implemented, this algorithm could be easily improved by accumulating more data on less prevalent injury categories. It is hereby possible to obtain a general overview of the phenomenon on the territory by monitoring its epidemiological evolution over time. This system could facilitate the timely activation of intervention policies, regardless of the alarming concentrations of injury events.

Moreover, it is also important to improve surveillance systems using classified ED data integrated with hospital discharge or mortality records to design effective injury prevention programs and interventions. In this general context, the proposed ML-based injury classification tool could be a first step toward addressing the burden of pediatric injuries from a new holistic perspective [36].

### Limitations

One first limitation of this study is that the data used for injury classification provide little information on what happens between ED admission and the final diagnosis. Moreover, as the triage service is extremely operator dependent, human factors represent an important confounding aspect of injury classification.

Another possible limitation is the small prevalence of certain types of injury, such as intentional injuries and drowning (among unintentional ones); this issue makes the algorithm’s performance on these types of events lacking. The injury and unintentional injury classifications constitute the leading classification task for this research; however, further research developments are needed to enrich the diagnosis data on these types of injuries and train the classification machine for a more refined surveillance tool. Moreover, the poorly represented classes of unintentional injuries constitute an issue to be deepened from a technical standpoint. Within this framework, further research is needed to develop algorithms tailored to handle severe class imbalance.

Another point to explore is the generalization of the algorithm; the MLT performance may be influenced by a training process performed on diagnoses data retrieved from the same center, where the referring physicians could maintain the same writing style across the data set. For this reason, data from other centers would be needed to generalize the validity of the epidemiological tool. Despite this limitation, this tool constitutes a proof of concept of an epidemiological surveillance attempt performed using a machine trained on data from a large pediatric ED referral center in Northeast Italy.

### Conclusions

This research paper reports an MLT-based free-text classification application conducted for the epidemiological surveillance of pediatric injuries. The algorithms have been trained considering the free-text diagnoses data of the Padova University Hospital ED unit, a large referral center in Northeast Italy.

The results of this study, for the injury classification task, showed that MLTs are a promising tool for improving epidemiological surveillance, allowing for the characterization of pediatric injuries in the ED by considering the free-text diagnoses as data sources.

The reported classification performance is satisfactory, especially for general injuries and intentional injury classification. These research results could facilitate the surveillance of a phenomenon that is often not easy to identify. Moreover, the approach could save time for health professionals working in the ED in manually classifying diagnoses for research purposes.

## Appendix

Multimedia Appendix 1 Supplementary figures.

## Abbreviations

DT: decision tree
ED: emergency department
GBM: gradient boosting method
MLT: machine learning technique
RF: random forest
SVM: support vector machine
WHO: World Health Organization

## Data Availability

The database is provided from an administrative data source of the University Hospital of Padua. They could be made available upon motivated request to the authors.
