# Supplementary material for: Pediatric Injury Surveillance From Uncoded Emergency Department Admission Records in Italy: Machine Learning–Based Text-Mining Approach
Source: JMIR Public Health Surveill. 2023 Jul 12;9:e44467. doi: 10.2196/44467 (PMC10372563; doi:10.2196/44467)
Supplement: Multimedia Appendix 1 [file publichealth_v9i1e44467_app1.docx]

Supplementary Material


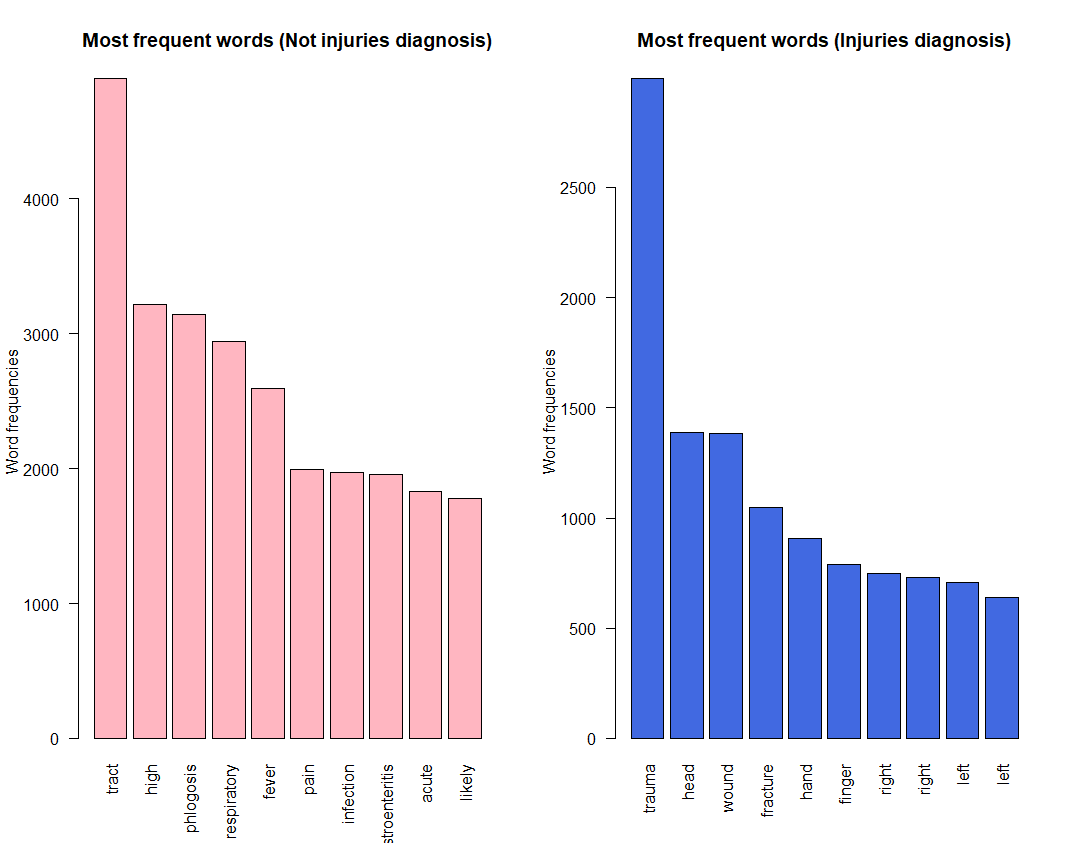


Figure S1 Injury vs. noninjury classification task. Word frequencies (absolute values, 10 most frequent terms) are shown according to the noninjuries and injuries in the gold standard cases.


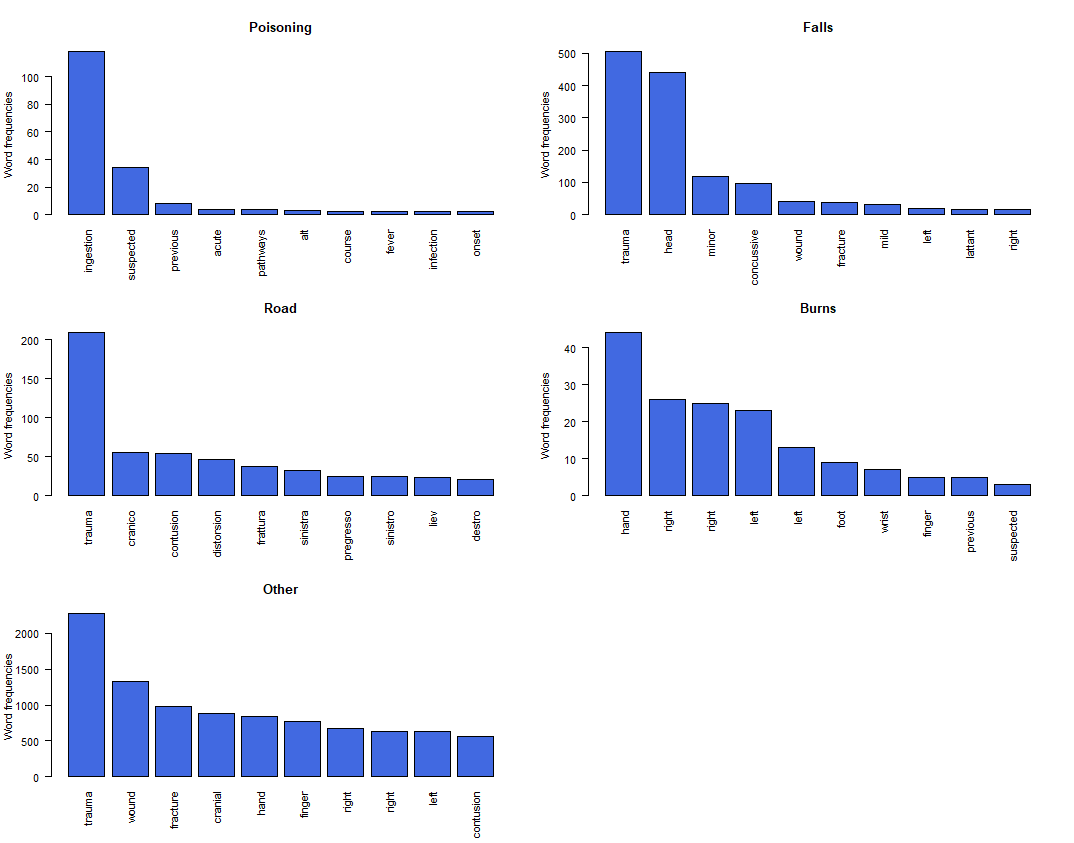


**Figure S2 Unintentional injury classification task. Word frequencies (absolute values, 10 most frequent terms) are shown according to the unintentional injury categories in the gold standard cases.**

Table S1 Disagreement in gold standard cross-validated predictions of the RF and SVM methods classified according to manually classified injury/noninjury methods. Continuous data are reported as medians (quartiles I and III); categorical data are reported as percentages and absolute frequencies. Wilcoxon-type tests were performed for continuous variables; the Pearson Chi-square test, or Fisher’s exact test, as appropriate, were performed for categorical variables.

| **Variable** | **Injury** | **Not Injury** | **Combined** | **Test** |
| --- | --- | --- | --- | --- |
|  | *(N=365)* | *(N=1263)* | *(N=1628)* |  |
| PREDICTIONS |  |  |  |  |
| **RF predictions:** injury | *52% (189)* | *51% (646)* | *51% (835)* | *P=.831* |
| noninjury | *48% (176)* | *49% (617)* | *49% (793)* |  |
| **SVM predictions**: injury | *48% (176)* | *49% (617)* | *49% (793)* | *P=.831* |
| noninjury | *52% (189)* | *51% (646)* | *51% (835)* |  |
| CHARACTERISTICS |  |  |  |  |
| **Age class:** 1-28 days | *1% ( 3)* | *13% (165)* | *10% (168)* | *P<.001* |
| 29 days – one year | *8% (28)* | *22% (278)* | *19% (306)* |  |
| 1-3 years | *19% (69)* | *17% (214)* | *17% (283)* |  |
| 4-5 years | *18% (65)* | *18% (228)* | *18% (293)* |  |
| 6-10 years) | *25% (91)* | *14% (183)* | *17% (274)* |  |
| 11-15 years | *30% (108)* | *11% (141)* | *15% (249)* |  |
| 16 years + | *0% ( 1)* | *4% (54)* | *3% (55)* |  |
| **Gender:** Female | *49% (180)* | *48% (607)* | *48% (787)* | *P=.673* |
| Male | *51% (185)* | *52% (656)* | *52% (841)* |  |
| **Nationality:** Other | *32% (117)* | *41% (515)* | *39% (632)* | *P=.003* |
| Italian | *68% (248)* | *59% (748)* | *61% (996)* |  |
| **Season:** Spring | *25% (93)* | *23% (289)* | *23% (382)* | *P=.009* |
| Summer | *29% (105)* | *26% (329)* | *27% (434)* |  |
| Winter | *18% (66)* | *27% (338)* | *25% (404)* |  |
| Fall | *28% (101)* | *24% (307)* | *25% (408)* |  |

Table S2 Unintentional injury classification task: comparative cross-validated performance measures of the MLT methods.

| *Poisoning performance*   \|  \| rf \| DT \| gbm \| svm \| \| --- \| --- \| --- \| --- \| --- \| \| Sensitivity \| 0.4432 \| 0.3201 \| 0.642 \| 0.6705 \| \| Specificity \| 0.9967 \| 0.9981 \| 0.9964 \| 0.9958 \| \| Pos Pred Value \| 0.7358 \| 0.7824 \| 0.789 \| 0.7712 \| \| Neg Pred Value \| 0.9884 \| 0.9859 \| 0.9925 \| 0.9931 \| \| Balanced Accuracy \| 0.7199 \| 0.6591 \| 0.8192 \| 0.8331 \|   *Falls performance*   \|  \| rf \| DT \| gbm \| svm \| \| --- \| --- \| --- \| --- \| --- \| \| Sensitivity \| 0.0753 \| 0 \| 0.0683 \| 0.0255 \| \| Specificity \| 0.9917 \| 1 \| 0.9935 \| 0.999 \| \| Pos Pred Value \| 0.4006 \| 0 \| 0.4351 \| 0.6522 \| \| Neg Pred Value \| 0.9356 \| 0.9313 \| 0.9353 \| 0.9329 \| \| Balanced Accuracy \| 0.5335 \| 0.5 \| 0.5309 \| 0.5122 \|   *Road traffic performance*   \|  \| rf \| DT \| gbm \| svm \| \| --- \| --- \| --- \| --- \| --- \| \| Sensitivity \| 0.0577 \| 0 \| 0.0068 \| 0.0038 \| \| Specificity \| 0.9928 \| 1 \| 0.999 \| 0.9996 \| \| Pos Pred Value \| 0.3409 \| 0 \| 0.3137 \| 0.4 \| \| Neg Pred Value \| 0.9423 \| 0.9393 \| 0.9397 \| 0.9395 \| \| Balanced Accuracy \| 0.5252 \| 0.5 \| 0.5029 \| 0.5017 \|   *Burns performance*   \|  \| rf \| DT \| gbm \| svm \| \| --- \| --- \| --- \| --- \| --- \| \| Sensitivity \| 0.1014 \| 0 \| 0.0195 \| 0 \| \| Specificity \| 0.9975 \| 1 \| 0.9996 \| 0.9998 \| \| Pos Pred Value \| 0.4876 \| 0 \| 0.5397 \| 0 \| \| Neg Pred Value \| 0.9796 \| 0.9774 \| 0.9778 \| 0.9774 \| \| Balanced Accuracy \| 0.5495 \| 0.5 \| 0.5095 \| 0.4999 \|   *Other unintentional injury performance*   \|  \| rf \| DT \| gbm \| svm \| \| --- \| --- \| --- \| --- \| --- \| \| Sensitivity \| 0.9783 \| 0.9973 \| 0.9878 \| 0.9961 \| \| Specificity \| 0.2884 \| 0.1688 \| 0.2815 \| 0.2636 \| \| Pos Pred Value \| 0.8313 \| 0.8114 \| 0.8313 \| 0.829 \| \| Neg Pred Value \| 0.7873 \| 0.9461 \| 0.8657 \| 0.9499 \| \| Balanced Accuracy \| 0.6333 \| 0.5831 \| 0.6347 \| 0.6299 \| |
| --- | --- | --- | --- | --- | --- | --- | --- | --- | --- | --- | --- | --- | --- | --- | --- | --- | --- | --- | --- | --- | --- | --- | --- | --- | --- | --- | --- | --- | --- | --- | --- | --- | --- | --- | --- | --- | --- | --- | --- | --- | --- | --- | --- | --- | --- | --- | --- | --- | --- | --- | --- | --- | --- | --- | --- | --- | --- | --- | --- | --- | --- | --- | --- | --- | --- | --- | --- | --- | --- | --- | --- | --- | --- | --- | --- | --- | --- | --- | --- | --- | --- | --- | --- | --- | --- | --- | --- | --- | --- | --- | --- | --- | --- | --- | --- | --- | --- | --- | --- | --- | --- | --- | --- | --- | --- | --- | --- | --- | --- | --- | --- | --- | --- | --- | --- | --- | --- | --- | --- | --- | --- | --- | --- | --- | --- | --- | --- | --- | --- | --- | --- | --- | --- | --- | --- | --- | --- | --- | --- | --- | --- | --- | --- | --- | --- | --- | --- | --- | --- | --- |

**Table S3 Characteristics of ED visits involving Padova-resident children. Continuous data are reported as medians (quartiles I and III); categorical data are reported as percentages and absolute frequencies.**

| **Variable** | **Padua resident ED visits** |
| --- | --- |
|  | (N=221175) |
| **Age year** | 1/4/9 |
| **Nationality**: Other | 22% (48598) |
| Italian | 78% (172577) |
| **Gender**: Female | 44% (96562) |
| Male | 56% (124613) |
| **Year** : 2007 | 8% (18005) |
| 2008 | 8% (18515) |
| 2009 | 8% (18490) |
| 2010 | 8% (18245) |
| 2011 | 8% (18473) |
| 2012 | 8% (17662) |
| 2013 | 8% (17306) |
| 2014 | 8% (17853) |
| 2015 | 8% (17005) |
| 2016 | 8% (17613) |
| 2017 | 9% (20622) |
| 2018 | 10% (21386) |

**Table S4 ED predicted injury classes according to the MLTs and observed gold standard cases. Relative percentages and absolute frequencies across different MLT methods and classification tasks are reported. Prediction intervals in percentages were also computed considering the MLT error rate (1-classification accuracy).**

|  |  |  |
| --- | --- | --- |
| ***Observed Gold Standard cases***  *injury* |  | 21% (8232) |
| *noninjury* |  | 79% (31344) |
| ***MLTs Predictions on ED*** |  |  |
| ***RF:*** *noninjury* |  | 75% [72; 78] (166682) |
| ***injury*** |  | 25% [22; 28] (54493) |
| ***DT:*** *noninjury* |  | 85% [79; 90] (187000) |
| *injury* |  | 15% [10; 21] (34175) |
| ***GBM:*** *noninjury* |  | 77% [74; 80] (169555) |
| *injury* |  | 23% [20; 26] (51620) |
| ***SVM:*** *noninjury* |  | 77% [74; 80] (169977) |
| *injury* |  | 23% [20; 26] (51198) |


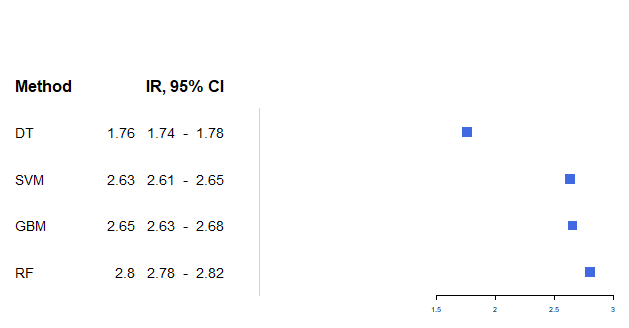


Figure S4 Incidence Rates (IR) per 100 Person-Time of ED visits for injuries for Padova resident children (0-18 years). The Person Time are 1,945,323 cumulated in the period 2007-2018. The number of cases in the period was estimated using the RF, GBM, DT, and SVM algorithms.
